# Implementation of an educational video about testicular torsion improves patient and caregiver awareness

**DOI:** 10.1038/s41390-025-04463-6

**Published:** 2025-09-29

**Authors:** Benjamin Abelson, Viktor Flores, Cyrus Adams, Mark Adams, John Brock, John C. Pope, John Thomas, Douglass B. Clayton, Alice Rothman, Abby S. Taylor

**Affiliations:** 1 Division of Pediatric Urology, Monroe Carell Jr. Children’s Hospital at Vanderbilt, Nashville, TN USA; 2 Department of Pediatrics, Monroe Carell Jr. Children’s Hospital at Vanderbilt, Nashville, TN USA; 3 Present Address: Phoenix Children’s, Phoenix, AZ USA; 4 Present Address: NYU Langone Health, New York, NY USA

## Abstract

**Background:**

Testicular torsion is an emergency that can require orchiectomy if too much time passes between ischemia onset and surgery.

**Methods:**

This prospective, survey-based study demonstrates that there is poor understanding about torsion among boys, parents, and school nurses, and that a short, animated video could improve this knowledge gap and motivate early presentation to the hospital. After completing a survey about managing acute testicular pain, participants viewed a video (https://share.vidyard.com/watch/7aKDRapNbeKrVzhdYrxton) and answered follow-up questions.

**Results:**

670 parents, 376 school nurses, 43 boys, and 47 pediatricians completed the study. 45% of parents have never heard of torsion. Over half of the boys report they would wait before telling a parent about testicular pain. Less than half of school nurses say they would recommend a child’s parents take him to the emergency department.

**Conclusions:**

Viewing an educational video improves awareness among boys, parents and school nurses that acute testicular pain needs to be evaluated and treated emergently.

**Impact:**

- Testicular torsion is the most common urologic emergency in children, but most boys and their caregivers are unaware of the diagnosis. This leads to delays in care and higher rates of testicular loss.
- Many hospital initiatives have sought to decrease delays for boys with testicular torsion after they interact with the healthcare system, however, most time delay occurs *prior* to arrival at the hospital.
- This is the first study to demonstrate that patient and parent education can be more effective in taking efficient care of boys with acute testicular torsion.

## Introduction

Acute testicular torsion (ATT) is a painful emergent condition in which the blood flow to the testicle is interrupted due to twisting of the spermatic cord, and if identified promptly, torsion can be managed with surgical correction, called orchiopexy. One in 1500 males is predicted to undergo surgery for torsion prior to age 18, but unfortunately, 30–40% of surgical interventions require removal of a testicle (orchiectomy) due to irreversible ischemic damage.^1,2^ Testicular salvage is dependent on timely evaluation and management, ideally in the first 6–8 h after the onset of pain.^3,4^ Recent hospital-based interventions have attempted to decrease the time from arrival in the emergency department to the incision in the operating room.^5,6^ However, the most significant delays in care occur *prior* to hospital arrival, when prompt evaluation depends on a young boy being willing to discuss scrotal pain with a family member, guardian, school nurse, coach, or healthcare provider.^4^

Studies have shown that most parents and boys are unaware of testicular torsion and may delay seeking treatment for acute scrotal pain.^7–9^ The objectives of this study are to better understand the causes of pre-hospital delays in care for ATT and demonstrate that a brief educational video can significantly impact the potential behaviors of those individuals involved in caring for boys with testicular pain before their arrival to the hospital.

## Methods

In collaboration with patient educators, pediatricians, parents, and pediatric urologists, we developed a short, animated video about testicular torsion (https://share.vidyard.com/watch/7aKDRapNbeKrVzhdYrxton). The video follows a teen boy, Danny, through two scenarios as he experiences testicular pain: First, he ignores the pain at school and at home, and this delay results in testicular loss. Second, he tells a teacher in a timely manner, and visits the school nurse who recommends that he go to the emergency department, where prompt surgical intervention saves the testicle. By following Danny through his experience with acute onset-testicular pain, we hoped to convey the typical signs and symptoms of testicular torsion, the methods of evaluating testicular torsion, the possible outcomes for a patient, and, most importantly, the urgency of evaluating acute scrotal pain in order to avoid orchiectomy. The video script was developed in conjunction with our hospital’s patient education team to confirm that we met high standards for health communication. English and Spanish subtitles were included to reach a diverse patient population.

Four target groups were selected: Parents, school nurses, pediatricians and boys at risk for ATT. The study was approved by the Vanderbilt University Medical Center IRB (IRB #210493). Parents were recruited via ResearchMatch, school nurses were contacted through our state’s school nursing organization (Tennessee Department of Education), pediatricians were recruited via email from a local non-profit organization of pediatric healthcare researchers and providers (Cumberland Pediatric Foundation), and boys ages 9–18 years were recruited during visits to their local pediatricians’ offices. All participants provided their electronic written consent (or in the case of minors, parents provided consent), and completed a pre-video questionnaire (See supplemental material). Participants then viewed our animated educational video, created in collaboration with Smarter Medical Care (Berkley, CA), and then completed a follow-up survey.

Data analysis was performed using SAS. Statistical analyses performed included descriptive statistics and Exact McNemar Chi Square tests for pre- and post-video comparison of medically appropriate response. Statistical significance was determined by a threshold *p*-value < 0.05.

## Results

### Parents

ResearchMatch surveys were completed by 670 parents. Eighty-two percent of the respondents were white, 71% were between 30 and 60 years old; and 60% of participants had children below age 20 (Table 1). Forty-six percent of the parents had completed graduate degrees, and 74.5% were privately insured. Thirty-one percent of the parents worked in the healthcare field. Of the respondents, 55% have heard of testicular torsion, and half of those individuals learned of torsion from their own medical training (Table 2). When asked, “If your son complained of sudden onset scrotal/groin/testicular pain when he is/was a pre-teen or teenager, what would be your first course of action?”, 22% of parents report they would go straight to the emergency room, while 20% report that they would wait for the pain to resolve on its own. The remainder would call their pediatrician. Of those respondents reporting that they would wait to see if the pain resolved on its own, 21% report that they would wait longer than 6 h. There was a statistically significant difference in the proportion of medically appropriate responses (proceed directly to the ED) to a scenario where their son developed acute testicular pain pre- and post-video intervention, *p* < 0.001 (Table 3).

**Table 1:** Demographic characteristics of parents surveyed.

Parental Demographics (*n* = 641)		
Race	Asian	12 (1.9%)
	Black/AA	46 (7.1%)
	Mixed	50 (7.8%)
	White	528 (82%)
Gender	Male	156 (24.2%)
	Female	485 (75.3%)
Highest Level of Education	High School	85 (13.2%)
	College/University	263 (40.7%)
	Graduate	298 (46.1%)
Health Insurance Status	Medicare/Medicaid	146 (22.6%)
	Private	483 (74.9%)
	Uninsured	13 (2.5%)
Employed in Healthcare Industry	Yes	198 (30.7%)
	No	443 (69.3%)

**Table 2:** Questionnaire results from parents regarding knowledge of testicular torsion and theoretical scenarios if their son were to develop acute scrotal pain.

Parent Survey Results		
Prior to video intervention, what would you do if your son complained of sudden onset scrotal pain as a pre-teen or teenager?	Call pediatrician Go straight to ED Wait to see if pain subsides	377 (58%) 139 (21.7%) 126 (19.6%)
Prior to video intervention, how long would you wait for the pain to go away? (of those who said they would wait)	< 1 h 1–3 h 3–6 h > 6 h	19 (15.1%) 56 (44.4%) 24 (19.9%) 27 (21.4%)
Have you ever heard of testicular torsion or “twisted testicle”?	Yes No	357 (55.3%) 288 (44.7%)
Have you ever discussed with your son sudden onset pain in his scrotum/testicle?	Yes No	91 (14.1%) 553 (85.9%)
After video intervention, what would you do If your son complained of sudden onset scrotal pain as a pre-teen or teenager?	Call pediatrician Go straight to ED Wait to see if subsides	125 (19.9%) 488 (77.7%) 15 (2.4%)
Did the video teach you something about testicular pain	Yes No	576 (91.7%) 52 (8.3%)
Would you show the video to male children in your household?	Yes No	564 (89.8%) 64 (10.2%)

**Table 3:** Pre- and post-video response to the following scenarios.

Pre-/Post-video Scenario Correct Response			
	Pre- Video Response	Post-Video Response	Chi Square (*p*-value)
Parents, # correct response (%)	138 (21.5%)	499 (77.8%)	**355.4** **(<0.0001)**
School nurse, # correct response (%)	162 (43.1%)	324 (86.2%)	**159** **(<0.0001)**
Boys, # correct response (%)	8 (28.6%)	27 (96.4%)	**19.0** **(<0.0001)**

Parents: “What would you do if your son complained of sudden onset scrotal/groin/testicular pain as a pre-teen or teenager?” ‘Correct’ response was ‘Go straight to ED.

School nurses: “What would you do if a student presents to you with acute scrotal pain?” ‘Correct’ response was ‘Call students’ caregiver and recommend they proceed to ED.

Boys: “If you developed sudden onset testicular pain, when would you get help?” ‘Correct’ response was ‘Immediately’

Exact McNemar’s Chi Square test to determine differences in providing the medically recommended/correct scenario responses pre- and post-video.

### School nurses

REDCap surveys were completed by 376 school nurses in response to the recruitment email distributed to approximately 2000 nurses through the Tennessee Department of Education. Twenty-seven percent of nurses have seen a boy with acute scrotal pain in their career. When asked what they would do if a student presented to their office with acute scrotal pain, 4% said they would ask the child to return to class and let them know if pain persists; 48% said they would call the patient’s caregiver for pickup and recommend they call their pediatrician; 43% report they would recommend the parent take the child straight to the emergency room. After watching the video 85% of the cohort after would recommend to parents that they take the child immediately to the ED and 90% found the video educational (Table 4). An exact McNemar’s test determined that there was a statistically significant difference in the proportion of the medically appropriate responses (direct caregiver to proceed to ED) to a scenario where a student presented to them with acute testicular pain pre- and post-video intervention, *p* = <0.0001 (Table 3).

**Table 4:** Questionnaire results from school nurse participants regarding knowledge of testicular torsion and theoretical scenarios if a student came to them with acute scrotal pain.

School nurse survey results		
Survey Question	Response	N
Have you seen a student with acute scrotal pain?	Yes No	102 (27.1%) 274 (72.9%)
Prior to video intervention, if a student presents to you with acute scrotal pain, what would be your next course of action?	Ask child to return to class Call student’s caregiver recommend they call PCP Call student’s caregiver recommend they go to ER Other	13 (3.5%) 181(48.1%) 162 (43.1%) 20 (5.3%)
After video intervention, if a student presents to you with acute scrotal pain, what would be your next course of action?	Ask child to return to class Call student’s caregiver recommend they call PCP Call student’s caregiver recommend they go to ER Other	2 (0.6%) 43 (11.9%) 310 (85.9%) 6 (1.7%)
Did you learn something from the video about testicular pain?	Yes No	323 (89.5) 38 (10.5%)

### Boys

There were 43 peri-pubertal and pubertal males that responded to the brochure with QR code posted in local pediatrician’s offices, of which 28 completed both the pre- and post-video surveys. The median age was 15 years (range 10-18), 60.7% were white, 17.9% were black, and 17.9% were Asian. Only 21.4% of boys reported having discussed testicular pain with their pediatrician in the past (Table 5). When presented with a pre-video scenario of developing sudden onset scrotal pain, 28.6% would seek help immediately, 53.6% would wait 4 h, and 17.9% would wait 8 h. After watching the video, 96.4% would get help immediately, and only 1 (3.6%) stated he would wait 4 h. Prior to the video, if the pain started at home, 78.6% would tell someone, though if it started at school, 21.4% would tell someone. After watching the video, 100% would tell someone at home and 82.1% would tell someone if they were in school. All respondents stated they learned something about ATT from watching the video and 85.7% would recommend the video to their friends (Table 5). An exact McNemar’s test determined that there was a statistically significant difference in the proportion of the medically appropriate responses (tell someone immediately) to a scenario where he developed acute testicular pain pre- and post-video intervention, *p* = <0.0001 (Table 3).

**Table 5:** Questionnaire results from boys regarding knowledge of scrotal pain and theoretical scenarios if they were to develop sudden onset testicular pain.

Boys Survey Results		
Survey Question	Response	N
Have you discussed testicular pain with pediatrician?	Yes No	6 (21.4%) 22 (78.6%)
Prior to video intervention: If you developed sudden onset testicular pain, when would you get help?	Immediately Wait 4 h Wait >8 h	8 (28.6%) 15 (53.6%) 5 (17.9%)
Prior to video intervention: If acute onset testicular pain occurred, I would tell someone:	At home At school	22 (78.6%) 6 (21.4%)
After video intervention: If you developed sudden onset testicular pain, when would you get help?	Immediately Wait 4 h Wait >8 h	27 (96.4%) 1 (3.6%) 0 (0%)
After video intervention: If you developed sudden onset testicular pain, when would you get help?	Immediately Wait 4 h Wait >8 h	27 (96.4%) 1 (3.6%) 0
Did the video teach you about testicular torsion?	Yes No	28 (100%) 0 (0%)
Would you recommend this video to your friends?	Yes No	24 (85.7%) 4 (14.3%)

### Pediatric Primary Care Providers

Forty-seven pediatric-specific primary care providers responded to the recruitment email and completed the survey, though 5 indicated that they were pediatric subspecialists who did not treat testicular pain and were excluded from the study. Of the 42 primary care providers, 83.3% were MD/DO and 16.7% were Advanced Practitioner Providers (APP) with 19% having less than 5 years, 19% with 5–10 years, 16.7% with 11–15 years, and 45.2% with over 15 years of practice experience. Only 38.1% of providers routinely counsel boys on scrotal pain at a median age of 11 years (4–13). In terms of the utility of a testicular torsion educational video, 85.7% would like to have an educational video available for counseling, 92.7% thought this video should be used as an educational tool for boys, 90.2% thought this video should be used as an educational tool for parents and guardians, and 73.2% would incorporate the video into their practice if it were available for distribution (Table 6).

**Table 6:** Questionnaire results from pediatric primary care provider participants regarding their practice patterns on scrotal pain and opinions on usefulness of a testicular torsion educational video for patients and families.

Pediatrician Survey Questions	Yes	No
Do you routinely counsel boys about scrotal pain? If yes, mean age	16 (38.1%) 11 yrs. (4–13)	26 (61.9%)
Would you like to have an educational video available?	36 (85.7%)	6 (14.3%)
Would you incorporate this video into your practice?	30 (73.2%)	11 (26.8%)
Should the video be used as an educational tool for boys?	38 (92.7%)	3 (7.3%)
Should the video be used as an educational tool for parents/guardians?	37 (90.2%)	4 (9.8%)

## Discussion

Boys with testicular torsion require prompt evaluation and treatment to avoid orchiectomy. Significant work has been done within the hospital setting to decrease the time from first interaction with the healthcare system (in the emergency department or outpatient clinic) to the beginning of a surgical procedure.^7,10^ In fact, U.S. News and World Report rankings for pediatric urology divisions include metrics for the speed of treatment of boys with testicular torsion, encouraging hospitals to initiate surgical treatment for torsion less than 4h  after registration in the emergency department.^11^ However, the majority of the time before surgery is spent at home or in school, *prior* to interaction with the healthcare system, and our study demonstrated that a short educational video is a useful tool in educating various stakeholders about testicular torsion.

This study corroborates prior work demonstrating that a minority of parents have even heard of acute testicular torsion.^7–9^ Without an improved understanding of the signs and symptoms of torsion by stakeholders outside of the healthcare system, and the risk of orchiectomy if treatment is delayed, we will be unable to decrease the rates of orchiectomy among boys with torsion. Though parents will likely be the first to know about their child’s testicular pain, education of school nurses, athletic coaches, teachers, and all pediatric healthcare providers has the potential to get children to interact with emergency care and urologists more efficiently. Worldwide efforts to recognize early stroke symptoms have proven effective, and although testicular torsion may confer less severe morbidity, our approach to reducing orchiectomy rates should be similar.^12^ Arevalo et al demonstrated that many institutions can get patients from the emergency room to the operating room in under an hour.^6^ However, if the majority of ischemia time occurs prior to arrival, and parents, boys, and other stakeholders do not have a familiarity with acute testicular torsion, continuing to focus on ER care pathways and efficient ultrasonography will prove futile.

We sought to develop a tool that is both educational and entertaining, to provide boys, their parents, and their caregivers a way to learn about testicular torsion amidst the endless other items that compete for their attention. As demonstrated in our survey, most pediatricians do not routinely counsel boys about testicular pain, and most boys have never discussed this with their doctor. Perhaps this video could be included in anticipatory guidance materials distributed as part of well-checks for boys who are at risk, or included in materials for a middle school health class.

Our study has several limitations that should be considered. There is certainly selection bias at play in our sample of parents, given the recruitment technique through ResearchMatch that means a significant portion of this group works in healthcare. However, we concluded that this population would, if anything, be “over-educated” about torsion compared to the general population. The school nurse, boy and pediatrician cohorts are geographically isolated to Middle Tennessee, and results may not necessarily be generalizable across the country. The cohort of boys at risk for torsion is small, a reflection of the challenges of recruiting boys and their parents to consent to a study on a potentially sensitive topic. Perhaps this is, in itself, a piece of evidence that we need to encourage boys to be open about discussing their genitourinary health.

## Conclusion

Boys who are risk of torsion, and the parents and school nurses who may initially field complaints of testicular pain, have limited awareness of the signs, symptoms and outcomes of this emergent medical condition. A short, animated video can serve to educate these cohorts, hopefully empowering boys to tell their caregivers about testicular pain immediately, and motivating their caregivers to seek emergency care. Decreasing the time from symptom onset to interaction with a healthcare provider is the next step to further decrease orchiectomy rates.

## Supplementary information

Supplementary information

## Data Availability

The datasets generated during and/or analysed during the current study are available from the corresponding author on reasonable request.
