# Supplementary material for: Implementation of an educational video about testicular torsion improves patient and caregiver awareness
Source: Pediatr Res. 2025 Sep 29;100(3):1166–70. doi: 10.1038/s41390-025-04463-6 (PMC13672084; doi:10.1038/s41390-025-04463-6)
Supplement: Supplementary file 1 [file 41390_2025_4463_MOESM1_ESM.docx]

**Instrument A – Boy survey**

- How old are you?
  - Free text
- What is your race/ethnicity?
  - White or Caucasian
  - Black or African American
  - Asian
  - American Indian or Alaska Native
  - Hispanic, Latino
  - Middle Eastern or North African
  - Native Hawaiian or Other Pacific Islander
  - Multiracial
  - Other: free text
- Zip code:
  - Free text
- Have you ever heard of “testicular torsion” or a “twisted testicle”?
  - Yes
  - No
- If you suddenly developed testicular pain at **HOME**, would you tell a parent or guardian?
  - Yes
  - No
- If you suddenly developed testicular pain at **SCHOOL**, would you tell a teacher or school nurse?
  - Yes
  - No
- If you suddenly developed testicular pain at home or at school, would you wait a few hours to see if it disappeared?
  - Yes
  - No
- How quickly do doctors recommend you go to the hospital for sudden testicular pain?
  - Immediately
  - Within 4 hours
  - Within 8 hours
  - Within 12 hours
  - Within 1-2 days
- A doctor or parent has discussed testicular pain with me in the past
  - True
  - False

Individual will then be given a link to a YouTube Video (5 min) and asked to return to the survey to answer the following questions:

- Did the video help teach you something about testicular torsion?
  - Yes
  - No
- How quickly do doctors recommend you go to the hospital for sudden testicular pain?
  - Immediately
  - Within 4 hours
  - Within 8 hours
  - Within 12 hours
  - Within 1-2 days
- If you suddenly developed testicular pain at **HOME**, would you tell a parent or guardian?
  - Yes
  - No
- If you suddenly developed testicular pain at **SCHOOL**, would you tell a teacher or school nurse?
  - Yes
  - No
- Would you recommend your friends see this video so they know about the importance of testicular torsion?
  - Yes
  - No

**Instrument C – Parent/guardian survey**

- Do you have a male child or are you a guardian of a male child?
  - If “no,” respondent disqualified from survey
  - If “yes,” continue survey
- What is your age (by group)?
  - <20
  - 20-29
  - 30-39
  - 40-49
  - 50-59
  - 60-69
  - > 70
- What is your son’s age group? Select all that apply
  - <5
  - 5-10
  - 11-15
  - 16-20
  - >20
- What is your race/ethnicity?
  - White or Caucasian
  - Black or African American
  - Asian
  - American Indian or Alaska Native
  - Hispanic, Latino
  - Middle Eastern or North African
  - Native Hawaiian or Other Pacific Islander
  - Multiracial
  - Other: free text
- What is your gender?
  - Male
  - Female
- What is the highest level of education you have completed?
  - High school or equivalent degree
  - College/university undergraduate degree
  - Graduate degree
- What is your insurance status?
  - Medicaid
  - Medicare
  - Private insurance
  - No insurance
- Do you work in healthcare?
  - Yes
  - No
- If your son complained of sudden onset scrotal/groin/testicular pain, what would be your first course of action?
  - Call your pediatrician
  - Go straight to the emergency room
  - Wait to see if it goes away
- If you selected, “wait to see if it goes away”, how long would you wait?
  - Less than 1 hour
  - 1-3 hours
  - 3-6 hours
  - More than 6 hours
  - N/A
- Have you ever heard of “testicular torsion,” or a “twisted testicle.”
  - Yes
  - No
- If you selected “yes, I have heard of testicular torsion”, who did you hear about it from?
  - My own medical education
  - Family or friend
  - My physician or my son’s physician
  - Other: Free text
- Have you ever discussed with your son(s) what they should do if they develop sudden scrotum/testicle pain?
  - Yes
  - No

The parent or guardian will then be asked to watch the YouTube video (5 minute) and then complete the following questions:

- Did you find the video informative?
  - Yes
  - No
- Now that you have watched the video, ff your son complained of sudden onset scrotal/groin/testicular pain, what would be your first course of action?
  - Call your pediatrician
  - Go straight to the emergency room
  - Wait to see if it goes away
- Would you show the video to the male children in your household?
  - Yes
  - No
- Do you have any comments or questions for the investigators and the creators of the video?
  - Free text

**Instrument D – School nurse survey**

- How long have you been practicing nursing?
  - 0-5 years
  - 5-10 years
  - 10-15 years
  - > 15 years
- How long have you practiced nursing in a school?
  - 0-5 years
  - 5-10 years
  - 10-15 years
  - > 15 years
- What are your credentials?
  - BSN or equivalent
  - MSN or equivalent
  - DNP
  - Advanced practice provider
- Have you ever seen a patient with acute scrotal pain in the school environment?
  - Yes
  - No
- Have you ever seen a patient with testicular torsion ?
  - Yes
  - No
  - Unsure
- If a student presents to you with acute scrotal pain, what would be your next step?
  - Ask the child to return to class and let you know if pain persists.
  - Call the patient’s caregiver for pickup and recommend they wait to see if the pain resolves.
  - Call the patient’s caregiver for pickup and recommend they go straight to the emergency room.
  - Call the patient’s caregiver for pickup and recommend they call their pediatrician.
  - Other: free text

The school nursing provider will then click a link to the YouTube video (5 min) and will be asked to answer the following questions afterwards.

- Do you think this video would be useful for pre-teen and teenage boys under your care?
  - Yes
  - No
- Do you think this video would be useful for parents or guardians of boys?
  - Yes
  - No
- Will this video impact how you care for patients with scrotal pain?
  - Yes
  - No

Do you have any comments or questions for the investigators and the creators of the video?

Free text

**Instrument B – General pediatrics healthcare provider survey**

- How long have you been practicing general pediatrics?
  - 0-5 years
  - 5-10 years
  - 10-15 years
  - > 15 years
- What are your credentials?
  - MD/DO in practice
  - MD/DO in training
  - Advanced practice provider
- Have you ever seen a patient with acute scrotal pain?
  - Yes
  - No
- Have you ever seen a patient with testicular torsion?
  - Yes
  - No
- If a patient calls your office with testicular pain during regular business hours, what is your typical practice?
  - Send patient directly to the emergency department
  - Send the patient directly to radiology and order an ultrasound
  - See the patient in clinic that day
  - Have the patient scheduled for the next available appointment

The healthcare provider will then click a link to the YouTube Video and will be asked to answer the following questions afterwards.

- Do you think this video would be useful for pre-teen and teenage boys under your care?
  - Yes
  - No
- Do you think this video would be useful for parents or guardians of boys?
  - Yes
  - No
- Will this video impact how you care for patients with scrotal pain?
  - Yes
  - No, I already knew about the urgent nature of testicular pain in boys
- Do you have any comments or questions for the investigators and the creators of the video?
  - Free text
